# 3-(4-Chloro­phen­yl)-2-methyl­acrylic acid

**DOI:** 10.1107/S1600536808022198

**Published:** 2008-07-19

**Authors:** Niaz Muhammad, Muhammad Nawaz Tahir, Zia-ur-Rehman, Saqib Ali, Farkhanda Shaheen

**Affiliations:** a Department of Chemistry, Quaid-i-Azam University, Islamabad 45320, Pakistan; b University of Sargodha, Department of Physics, Sargodha, Pakistan

## Abstract

In the crystal structure of the title compound, C_10_H_9_ClO_2_, dimers form as a result of inter­molecular O—H⋯O bonding. These dimers are linked to each other *via* C—H⋯O bonds, where the CH group belongs to the benzene ring and the O atom is from the carbonyl group of an adjacent mol­ecule. There exist two inter­molecular C—H⋯O hydrogen bonds, which individually form five-membered rings. There also exists a π–π inter­action between the aromatic ring and its symmetry counterpart, with a centroid–centroid distance of 4.0202 (17) Å, and a C—H⋯π inter­action between a methyl CH group and the aromatic ring.

## Related literature

For related literature, see: Bernstein *et al.* (1995 ▶); Bravo (1998 ▶); Burt (2004 ▶); Hertog *et al.* (1995 ▶); Muhammad *et al.* (2007*a* ▶,*b* ▶, 2008*a* ▶,*b* ▶); Muhammad, Ali *et al.* (2008 ▶); Niaz *et al.* (2008 ▶).
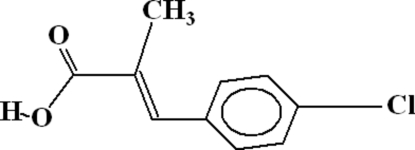


## Experimental

### 

#### Crystal data

C_10_H_9_ClO_2_
*M* *_r_* = 196.62
Triclinic, 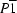

*a* = 7.2164 (6) Å
*b* = 8.2746 (7) Å
*c* = 9.1762 (8) Å
α = 115.182 (4)°
β = 108.022 (4)°
γ = 90.052 (5)°
*V* = 465.91 (7) Å^3^
*Z* = 2
Mo *K*α radiation
μ = 0.37 mm^−1^
*T* = 296 (2) K
0.28 × 0.20 × 0.18 mm

#### Data collection

Bruker Kappa APEXII CCD diffractometer
Absorption correction: multi-scan (*SADABS*; Bruker, 2005 ▶) *T*_min_ = 0.910, *T*_max_ = 0.930
7513 measured reflections
2692 independent reflections
1782 reflections with *I* > 2σ(*I*)
*R*_int_ = 0.029

#### Refinement

*R*[*F* ^2^ > 2σ(*F* ^2^)] = 0.059
*wR*(*F* ^2^) = 0.217
*S* = 1.10
2692 reflections
122 parameters
H atoms treated by a mixture of independent and constrained refinement
Δρ_max_ = 0.53 e Å^−3^
Δρ_min_ = −0.27 e Å^−3^

### 

Data collection: *APEX2* (Bruker, 2007 ▶); cell refinement: *APEX2*; data reduction: *SAINT* (Bruker, 2007 ▶); program(s) used to solve structure: *SHELXS97* (Sheldrick, 2008 ▶); program(s) used to refine structure: *SHELXL97* (Sheldrick, 2008 ▶); molecular graphics: *ORTEP-3 for Windows* (Farrugia, 1997 ▶) and *PLATON* (Spek, 2003 ▶); software used to prepare material for publication: *WinGX* (Farrugia, 1999 ▶) and *PLATON*.

## Supplementary Material

Crystal structure: contains datablocks global, I. DOI: 10.1107/S1600536808022198/bq2090sup1.cif

Structure factors: contains datablocks I. DOI: 10.1107/S1600536808022198/bq2090Isup2.hkl

Additional supplementary materials:  crystallographic information; 3D view; checkCIF report

## Figures and Tables

**Table 1:** Hydrogen-bond geometry (Å, °)

*D*—H⋯*A*	*D*—H	H⋯*A*	*D*⋯*A*	*D*—H⋯*A*
O1—H1⋯O2^i^	0.88 (4)	1.76 (4)	2.643 (3)	176.4 (14)
C3—H3*A*⋯O2	0.96	2.41	2.765 (4)	101
C4—H4⋯O1	0.93	2.32	2.720 (3)	106
C9—H9⋯O2^ii^	0.93	2.57	3.458 (3)	159
C3—H3a⋯*Cg*^iii^	0.96	2.84	3.638 (3)	141

Symmetry codes: (i) 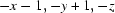; (ii) 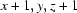; (iii) 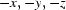. *Cg* is the centroid of the C5–C10 ring.

**Fig. 1.**
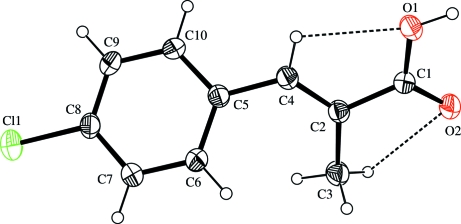
ORTEP drawing of (I) with the atom numbering scheme. The thermal ellipsoids are drawn at the 50% probability level. The intramolecular H-bonds are shown by doted lines.

**Fig. 2.**
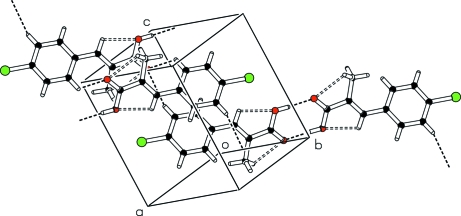
The packing figure (PLATON: Spek, 2003) which shows the dimeric nature of the compound and the interlinkages of the dimers.

## supplementary crystallographic information

### Comment

Cinnamic acids compose a relatively large family of organic acid isomers (Bravo, 1998). In nature, cinnamic acid derivatives are important metabolic building blocks in the production of lignins for higher plants. Cinnamic acid possesses antibacterial, antifungal and parasite fighting abilities (Burt, 2004). A derivative of cinnamic acid is an important pharmaceutical for high blood pressure, stroke prevention and possess antitumour activity (Hertog *et al.*, 1995). In continuation of our efforts to synthesize various derivatives of cinnamic acids (Niaz *et al.*, 2008, Muhammad, Ali *et al.*, 2008) and their tin complexes (Muhammad *et al.*, 2008*a*, 2008*b*), we herein report the structure of the title compound (I).

The crystal structure of 3-(4-Bromophenyl)-2-methylacrylic acid (II) (Muhammad *et al.*, 2007*a*) and 3-(4-Bromophenyl)-2-ethylacrylic acid (Muhammad *et al.*, 2007*b*) has been previously reported. The title compound (I) have a replacement of Br-atom with Cl-atom. Thus the reported compound (II) is the best example for the comparison of bond geometry *etc*.

In the crystal structure of the title compound, the C—C bonds are in the range [1.467 (3)–1.503 (4) Å], and C==C have a value of 1.341 (3) Å. The resonant C—O bonds have values of 1.231 (3) and 1.310 (3) Å. In the asymmetric unit, there are two intermolecular H-bonds of C—H···O type (Table 2, Fig 1). Due to these H-bonds two five membered rings (O1/C1/C2/C4/H4···O1) and (O2/C1/C2/C3/H3A···O2) are formed. Centrosymmetric dimers, *R*_2_^2^(8) (Bernstein *et al.* 1995) are formed due to the intermolecular O1—H1···O2^i^ [symmetry code: i = -*x* - 1, -*y* + 1, -*z*] hydrogen bonding. These dimers are linked to each other by intermolecular H-bonding, C9—H9···O2^ii^ [symmetry code: ii = *x* + 1, *y*, *z* + 1] as shown in Fig 2. There exist an interaction, C3—H3A···*Cg*^iii^ [symmetry code: iii = -*x*, -*y*, -*z*] with a distance of 3.638 (3) Å between C3 and *Cg*^iii^ [Cg is the center of the (C5-C10) benzene ring]. There also exist a π···π-interaction between the benzene rings of adjacent molecules. The distance between the centroids of *Cg* and *Cg*^iv^ [symmetry code: iv = -*x* + 1, -*y* + 1, -*z* + 1], is 4.0202 (17) Å.

### Experimental

Compound (I) was prepared according to the reported procedure (Muhammad *et al.*, 2007*a*). A mixture of 4-chlorobenzaldehyde (1.40 g, 10 mmol), methylmalonic acid (2.36 g, 20 mmol) and piperidine (1.98 ml, 20 mmol) in a pyridine (12.5 ml) solution was heated on a steam-bath for 24 h. The reaction mixture was cooled and added to a mixture of 25 ml of concentrated HCl and 50 g of ice. The precipitate formed in the acidified mixture was filtered off and washed with ice-cold water. The product was recrystallized from ethanol. The yield was 89%.

### Refinement

The coordinates of H atom attached to O1 were refined freely. All other H atoms were positioned geometrically, C—H = 0.93, and 0.96 Å for aromatic and methyl H, and constrained to ride on their parent atoms and were treated as isotropic with *U*_iso_(H) = *xU*_eq_(C,*O*), where *x* = 1.5 for methyl H, and *x* = 1.2 for all other H atoms.

### Crystal data

**Table:**

C_10_H_9_ClO_2_	*Z* = 2
*M**_r_* = 196.62	*F*_000_ = 204
Triclinic, *P*1	*D*_x_ = 1.402 Mg m^−^^3^
Hall symbol: -P 1	Mo *K*α radiation λ = 0.71073 Å
*a* = 7.2164 (6) Å	Cell parameters from 2692 reflections
*b* = 8.2746 (7) Å	θ = 2.6–30.3º
*c* = 9.1762 (8) Å	µ = 0.37 mm^−^^1^
α = 115.182 (4)º	*T* = 296 (2) K
β = 108.022 (4)º	Prism, colourless
γ = 90.052 (5)º	0.28 × 0.20 × 0.18 mm
*V* = 465.91 (7) Å^3^	

### Data collection

**Table:**

Bruker Kappa APEXII CCD diffractometer	2692 independent reflections
Radiation source: fine-focus sealed tube	1782 reflections with *I* > 2σ(*I*)
Monochromator: graphite	*R*_int_ = 0.029
Detector resolution: 7.2 pixels mm^-1^	θ_max_ = 30.3º
*T* = 296(2) K	θ_min_ = 2.6º
ω scans	*h* = −10→8
Absorption correction: multi-scan(SADABS; Bruker, 2005)	*k* = −10→11
*T*_min_ = 0.910, *T*_max_ = 0.930	*l* = −12→12
7513 measured reflections	

### Refinement

**Table:**

Refinement on *F*^2^	Secondary atom site location: difference Fourier map
Least-squares matrix: full	Hydrogen site location: inferred from neighbouring sites
*R*[*F*^2^ > 2σ(*F*^2^)] = 0.059	H atoms treated by a mixture of independent and constrained refinement
*wR*(*F*^2^) = 0.217	*w* = 1/[σ^2^(*F*_o_^2^) + (0.1083*P*)^2^ + 0.1931*P*] where *P* = (*F*_o_^2^ + 2*F*_c_^2^)/3
*S* = 1.10	(Δ/σ)_max_ < 0.001
2692 reflections	Δρ_max_ = 0.53 e Å^−^^3^
122 parameters	Δρ_min_ = −0.26 e Å^−^^3^
Primary atom site location: structure-invariant direct methods	Extinction coefficient: ?

### Special details

**Table:**

Geometry. Bond distances, angles *etc*. have been calculated using the rounded fractional coordinates. All su's are estimated from the variances of the (full) variance-covariance matrix. The cell e.s.d.'s are taken into account in the estimation of distances, angles and torsion angles
Refinement. Refinement of *F*^2^ against ALL reflections. The weighted *R*-factor *wR* and goodness of fit *S* are based on *F*^2^, conventional *R*-factors *R* are based on *F*, with *F* set to zero for negative *F*^2^. The threshold expression of *F*^2^ > σ(*F*^2^) is used only for calculating *R*-factors(gt) *etc*. and is not relevant to the choice of reflections for refinement. *R*-factors based on *F*^2^ are statistically about twice as large as those based on *F*, and *R*- factors based on ALL data will be even larger.

### Fractional atomic coordinates and isotropic or equivalent isotropic displacement parameters (Å^2^)

**Table:**

	*x*	*y*	*z*	*U*_iso_*/*U*_eq_	
Cl1	0.80795 (12)	0.09608 (15)	0.42599 (12)	0.0771 (4)	
O1	−0.2602 (3)	0.5461 (3)	0.1669 (2)	0.0510 (6)	
O2	−0.3959 (3)	0.3388 (3)	−0.1034 (2)	0.0543 (6)	
C1	−0.2554 (3)	0.4027 (3)	0.0322 (3)	0.0390 (7)	
C2	−0.0735 (3)	0.3196 (3)	0.0525 (3)	0.0371 (7)	
C3	−0.0680 (4)	0.1670 (4)	−0.1102 (3)	0.0484 (8)	
C4	0.0606 (3)	0.3764 (3)	0.2098 (3)	0.0403 (7)	
C5	0.2464 (3)	0.3096 (3)	0.2602 (3)	0.0364 (6)	
C6	0.3638 (4)	0.2508 (4)	0.1573 (3)	0.0432 (8)	
C7	0.5363 (4)	0.1864 (4)	0.2086 (3)	0.0461 (8)	
C8	0.5923 (4)	0.1793 (4)	0.3641 (3)	0.0438 (7)	
C9	0.4803 (4)	0.2379 (4)	0.4686 (3)	0.0493 (8)	
C10	0.3099 (4)	0.3051 (4)	0.4171 (3)	0.0451 (7)	
H1	−0.377 (5)	0.580 (5)	0.142 (5)	0.0612*	
H3A	−0.19861	0.10124	−0.17914	0.0725*	
H3B	0.01747	0.08750	−0.08293	0.0725*	
H3C	−0.01950	0.21487	−0.17241	0.0725*	
H4	0.03229	0.46997	0.29797	0.0484*	
H6	0.32524	0.25512	0.05263	0.0518*	
H7	0.61382	0.14812	0.13947	0.0554*	
H9	0.51873	0.23233	0.57269	0.0592*	
H10	0.23624	0.34816	0.48924	0.0541*	

### Atomic displacement parameters (Å^2^)

**Table:**

	*U*^11^	*U*^22^	*U*^33^	*U*^12^	*U*^13^	*U*^23^
Cl1	0.0555 (5)	0.1129 (8)	0.0698 (6)	0.0449 (5)	0.0181 (4)	0.0492 (5)
O1	0.0415 (10)	0.0576 (12)	0.0430 (10)	0.0207 (8)	0.0116 (8)	0.0147 (9)
O2	0.0436 (10)	0.0686 (13)	0.0391 (10)	0.0217 (9)	0.0075 (8)	0.0183 (9)
C1	0.0377 (11)	0.0459 (13)	0.0373 (12)	0.0117 (10)	0.0140 (9)	0.0213 (10)
C2	0.0357 (11)	0.0379 (12)	0.0411 (12)	0.0091 (9)	0.0141 (9)	0.0202 (10)
C3	0.0444 (13)	0.0477 (14)	0.0430 (13)	0.0116 (11)	0.0115 (11)	0.0138 (11)
C4	0.0365 (11)	0.0418 (12)	0.0395 (12)	0.0109 (9)	0.0122 (9)	0.0160 (10)
C5	0.0335 (10)	0.0351 (11)	0.0363 (11)	0.0060 (9)	0.0105 (9)	0.0131 (9)
C6	0.0397 (12)	0.0562 (15)	0.0408 (12)	0.0112 (10)	0.0143 (10)	0.0276 (12)
C7	0.0359 (11)	0.0604 (16)	0.0443 (13)	0.0130 (11)	0.0152 (10)	0.0245 (12)
C8	0.0349 (11)	0.0487 (14)	0.0413 (12)	0.0103 (10)	0.0060 (10)	0.0192 (11)
C9	0.0461 (13)	0.0627 (17)	0.0323 (12)	0.0133 (12)	0.0063 (10)	0.0200 (12)
C10	0.0412 (12)	0.0563 (15)	0.0310 (11)	0.0105 (11)	0.0116 (10)	0.0141 (11)

### Geometric parameters (Å, °)

**Table:**

Cl1—C8	1.734 (3)	C7—C8	1.385 (4)
O1—C1	1.310 (3)	C8—C9	1.376 (4)
O2—C1	1.231 (3)	C9—C10	1.382 (4)
O1—H1	0.88 (4)	C3—H3A	0.9600
C1—C2	1.480 (3)	C3—H3B	0.9600
C2—C3	1.503 (4)	C3—H3C	0.9600
C2—C4	1.341 (3)	C4—H4	0.9300
C4—C5	1.467 (3)	C6—H6	0.9300
C5—C10	1.386 (4)	C7—H7	0.9300
C5—C6	1.397 (4)	C9—H9	0.9300
C6—C7	1.381 (4)	C10—H10	0.9300
			
C1—O1—H1	109 (3)	C5—C10—C9	121.4 (2)
O1—C1—O2	121.8 (2)	C2—C3—H3A	109.00
O1—C1—C2	116.5 (2)	C2—C3—H3B	109.00
O2—C1—C2	121.7 (2)	C2—C3—H3C	109.00
C1—C2—C4	118.9 (2)	H3A—C3—H3B	109.00
C3—C2—C4	126.8 (2)	H3A—C3—H3C	109.00
C1—C2—C3	114.2 (2)	H3B—C3—H3C	110.00
C2—C4—C5	128.1 (2)	C2—C4—H4	116.00
C4—C5—C10	118.9 (2)	C5—C4—H4	116.00
C6—C5—C10	118.1 (2)	C5—C6—H6	119.00
C4—C5—C6	122.9 (2)	C7—C6—H6	119.00
C5—C6—C7	121.0 (2)	C6—C7—H7	120.00
C6—C7—C8	119.2 (3)	C8—C7—H7	120.00
Cl1—C8—C7	118.7 (2)	C8—C9—H9	120.00
Cl1—C8—C9	120.3 (2)	C10—C9—H9	120.00
C7—C8—C9	120.9 (3)	C5—C10—H10	119.00
C8—C9—C10	119.2 (3)	C9—C10—H10	119.00
			
O1—C1—C2—C3	−174.4 (2)	C10—C5—C6—C7	1.3 (5)
O1—C1—C2—C4	9.9 (4)	C4—C5—C10—C9	177.8 (3)
O2—C1—C2—C3	6.7 (4)	C6—C5—C10—C9	−2.5 (5)
O2—C1—C2—C4	−169.1 (3)	C5—C6—C7—C8	0.4 (5)
C1—C2—C4—C5	177.8 (3)	C6—C7—C8—Cl1	179.4 (3)
C3—C2—C4—C5	2.6 (5)	C6—C7—C8—C9	−1.0 (5)
C2—C4—C5—C6	35.4 (4)	Cl1—C8—C9—C10	179.5 (3)
C2—C4—C5—C10	−145.0 (3)	C7—C8—C9—C10	−0.2 (5)
C4—C5—C6—C7	−179.0 (3)	C8—C9—C10—C5	2.0 (5)

### Hydrogen-bond geometry (Å, °)

**Table:**

*D*—H···*A*	*D*—H	H···*A*	*D*···*A*	*D*—H···*A*
O1—H1···O2^i^	0.88 (4)	1.76 (4)	2.643 (3)	176.4 (14)
C3—H3A···O2	0.96	2.41	2.765 (4)	101
C4—H4···O1	0.93	2.32	2.720 (3)	106
C9—H9···O2^ii^	0.93	2.57	3.458 (3)	159
C3—H3a···Cg^iii^	0.96	2.84	3.638 (3)	141

Symmetry codes: (i) −*x*−1, −*y*+1, −*z*; (ii) *x*+1, *y*, *z*+1; (iii) −*x*, −*y*, −*z*.
